# Biomarkers of Neonatal Sepsis: Where We Are and Where We Are Going

**DOI:** 10.3390/antibiotics12081233

**Published:** 2023-07-26

**Authors:** Giovanni Boscarino, Rossana Migliorino, Giulia Carbone, Giusy Davino, Valentina Giovanna Dell’Orto, Serafina Perrone, Nicola Principi, Susanna Esposito

**Affiliations:** 1 Pediatric Clinic, Department of Medicine and Surgery, University of Parma, 43126 Parma, Italy; giovanni.boscarino@unipr.it (G.B.); antonellarossana.migliorino@unipr.it (R.M.); giulia.carbone@unipr.it (G.C.); giusy.davino@unipr.it (G.D.); 2 Neonatal Unit, Department of Medicine and Surgery, University of Parma, 43126 Parma, Italy; valentinagiovanna.dellorto@unipr.it (V.G.D.); serafina.perrone@unipr.it (S.P.); 3 Università degli Studi di Milano, 20122 Milan, Italy; nicola.principi@unimi.it

**Keywords:** biomarker, early onset sepsis, late onset sepsis, neonatal sepsis, neonatal infections

## Abstract

Neonatal sepsis is a bacterial bloodstream infection leading to severe clinical manifestations frequently associated with death or irreversible long-term deficits. Antibiotics are the drug of choice to treat sepsis, regardless of age. In neonates, the lack of reliable criteria for a definite diagnosis and the supposition that an early antibiotic administration could reduce sepsis development in children at risk have led to a relevant antibiotic overuse for both prevention and therapy. The availability of biomarkers of neonatal sepsis that could alert the physician to an early diagnosis of neonatal sepsis could improve the short and long-term outcomes of true sepsis cases and reduce the indiscriminate and deleterious use of preventive antibiotics. The main aim of this narrative review is to summarize the main results in this regard and to detail the accuracy of currently used biomarkers for the early diagnosis of neonatal sepsis. Literature analysis showed that, despite intense research, the diagnosis of neonatal sepsis and the conduct of antibiotic therapy cannot be at present decided on the basis of a single biomarker. Given the importance of the problem and the need to reduce the abuse of antibiotics, further studies are urgently required. However, instead of looking for new biomarkers, it seems easier and more productive to test combinations of two or more of the presently available biomarkers. Moreover, studies based on omics technologies should be strongly boosted. However, while waiting for new information, the use of the clinical scores prepared by some scientific institutions could be suggested. Based on maternal risk factors and infant clinical indicators, sepsis risk can be calculated, and a significant reduction in antibiotic consumption can be obtained.

## 1. Introduction

Neonatal sepsis is a bacterial bloodstream infection leading to severe clinical manifestations frequently associated with death or irreversible long-term deficits. Death can occur in 3–4% and up to 24% of neonates born in industrialized countries [1] and in the developing world [2], respectively. Among survivors, adverse neurodevelopmental outcomes at follow-up, including cerebral palsy, reduced mental and psychomotor development, and vision impairment are the most long-term deficits [3].

According to most experts, neonatal sepsis is categorized as early onset sepsis (EOS) if diagnosed in the first 72 h after birth or as late onset sepsis (LOS) if diagnosed after this period [4]. EOS is mainly due to vertical transmission of *Escherichia coli* and Group B *Streptococcus* from women with chorioamnionitis, prolonged rupture of membranes, and GBS colonization. LOS is often caused by pathogens acquired nosocomially in neonates at risk because of prematurity, presence of invasive instrumentation, use of parenteral nutrition, and mechanical ventilation [5,6]. Data concerning the epidemiology of neonatal sepsis differ significantly according to the criteria used to define the disease [4]. However, it has been calculated that in industrialized countries, incidence of EOS and LOS is no lower than 0.3–0.8 cases/1000 live births and about 6 cases/1000 live births, respectively [7,8]. Significantly higher values, up to several dozen/1000 live births, have been calculated for developing countries [9]. Together with the country of birth, several other factors influence the risk of neonatal sepsis development. Among these, birth weight (BW) and gestational age (GA) are two factors that are inversely associated with neonatal sepsis occurrence. In very low birth weight (VLBW) neonates, even in industrialized countries, rates of EOS and LOS increase to 20/1000 and 200/1000, respectively [10]. Similarly, 36.3% of neonates with a GA < 28 weeks have at least one episode of LOS, as compared with 29.6%, 17.5%, and 16.5% of those with a GA of 29–32 weeks, 33–36 weeks, and term infants [11].

Diagnosis of neonatal sepsis, especially of EOS, can be very difficult on the basis of clinical findings. In adults and in older children, sepsis is defined as a life-threatening organ dysfunction caused by a dysregulated host response to infection. Presence and severity of organ dysfunction is established using validated scoring systems that identify and quantify abnormalities according to clinical findings, laboratory data, or therapeutic measures [12]. Unfortunately, this definition cannot be applied to neonates as several studies have shown that while using criteria prepared for adults and older children, a great number of documented neonatal sepsis cases were not identified. In a study involving 476 term neonates, the identification of EOS was possible only in 53% of enrolled infants [13]. A greater number of sepsis cases are lost when preterm neonates are studied [14]. Several factors explain why criteria used to define sepsis in adults do not apply to neonates. Neonates, the greater the prematurity, the more likely the neonate is to have an immature immune system [15]. This leads to an increased risk of infection and to a different inflammatory and clinical response to any infectious agent. Moreover, reactions of neonates to harmful stimuli are quite similar; regardless, they are infectious, metabolic, or traumatic [16]. Early-stage symptoms of sepsis in neonates are subtle and non-specific and frequently common to other conditions. Respiratory problems, bradycardia, cyanosis, and temperature instability are described in infants with sepsis but can be found in neonates as an index of poor adaptation to extrauterine life or as signs of a non-infectious disease [17]. Because of this, the definition of sepsis in neonates is still lacking, and several scientific institutions have suggested specific criteria for the proper identification of sepsis in neonates. In most cases, together with child conditions, specific parameters including local epidemiology, GA, and several maternal characteristics are considered to prepare a risk calculator that is used to decide which children should be treated [18,19].

Antibiotics are the drug of choice to treat sepsis, regardless of age. In neonates, the lack of reliable criteria for a definite diagnosis and the supposition that early antibiotic administration could reduce sepsis development in children at risk have led to a relevant antibiotic overuse for both prevention and therapy [20,21]. Since the beginning of the antibiotic era, in most hospitals, all neonates at risk of infection, including most preterm infants, were given large spectrum antibiotics, even in the absence of a clinical manifestation suggesting infectious disease [22]. Despite this negative, prescriptive attitudes have been partially reduced by the introduction, at least in some hospitals, of specific stewardship programs [23,24], but antibiotic overuse in neonates still persists and is associated with several relevant problems [25,26]. It favors emergence of antimicrobial resistance and promotes dysbiosis, which has been associated with the development of life-long unwanted health problems, such as obesity, type I diabetes, asthma, autism spectrum disorders, necrotizing enterocolitis, and earlier death [27]. The availability of markers of neonatal sepsis that could address the physician to an early diagnosis of neonatal sepsis could improve the short and long-term outcomes of true sepsis cases and reduce the indiscriminate and deleterious use of preventive antibiotics. In the last 30 years, several attempts to identify biomarkers of neonatal sepsis have been made. The main aim of this narrative review is to summarize the main results in this regard and to detail the accuracy of currently used biomarkers for the early diagnosis of neonatal sepsis. To this end, we conducted electronic research in the PubMed database using “neonatal sepsis” “AND biomarkers” OR “blood culture” OR “blood cell count” OR “immature-to-total neutrophil ratio” OR “platelet count” OR “c-reactive protein” or “CRP” or “procalcitonin” or “PCT” or “amyloid A” OR “proadrenomelullin” OR “inflammatory markers” OR “cytokine“ or “interleukin-6” or “Interleukin-8” OR “tumor necrosis factor” OR “presepsin” OR “soluble triggering receptor” OR “sTREM-1” OR “cluster differentiation molecule-64” OR “CD-64” OR “omics” as keywords. Only articles written in English were selected, and a manual search of the references of eligible articles was made.

## 2. Characteristics of an Ideal Biomarker of Neonatal Sepsis

For a long time, the characteristics of an ideal marker for the early identification of children with EOS or LOS have been precisely defined [28]. It has been established that an ideal marker should rapidly increase after disease onset and equally rapidly decrease once the infection has been cured. It should have high sensitivity (~100%) and specificity (>85%) in the diagnosis of neonatal sepsis, with a high negative predictive value (~100%) and positive predictive value (>85%). Moreover, it should provide reliable information on when to start and when to stop antibiotic therapy in order to reduce antibiotic overuse, contain the development of bacterial resistance, and avoid significant modification of gut microbiota. Maternal, perinatal, or postnatal factors should not influence its physiologic kinetic. Finally, methods for marker detection should be easy to perform, comparable between different laboratories, require very small amounts of the sample, and be cost-effective [28].

## 3. Biomarkers Presently Used in Clinical Practice

### 3.1. Hematological Biomarkers

#### 3.1.1. Blood Culture

As neonatal sepsis is the consequence of a bacterial infection, traditionally, a positive blood culture is considered the gold standard for the diagnosis of this disease. However, blood cultures have a long turnaround time (TAT) and very low sensitivity that contribute to inappropriate antibiotic therapy. About 70% of septic neonates have low-colony-count bacteremia that result in negative cultures [29]. Moreover, it requires an invasive procedure to draw blood. Finally, results are strongly conditioned by the inoculant volume. The recommended minimal blood volume for the culture in newborn infants is 1 mL, but it has been found that up to 60% of sample volumes in clinical practice are limited to 0.5 mL, leading to a negative test [30]. These findings highlight that a blood culture is not appropriate for the diagnosis of neonatal sepsis. Important advances can be made using molecular methods, such as a polymerase chain reaction (PCR), real-time PCR, pyrosequencing, and microfluidic technology [31]. The availability of reliable results is significantly accelerated from days to hours. Sensitivity is significantly increased. In a meta-analysis of 23 studies comparing traditional blood cultures to molecular methods, it was calculated that the sensitivity and specificity of PCR assays performed the best with 96% sensitivity and 96% specificity [32]. However, molecular methods devoted to bacterial identification do not allow for one to know the antibiotic susceptibility of the infecting pathogen. Moreover, they require specialized biology laboratories and special equipment, as well, that are not available in many hospital settings, particularly in the third world.

#### 3.1.2. White Blood Cell Count, Absolute Neutrophil Count, Immature-to-Total Neutrophil Ratio and Platelet Count

A great number of studies have evaluated the role of white blood cell count (WBC), absolute neutrophil count (ANC), immature-to-total neutrophil ratio (I/T), and platelet count as potential markers of neonatal sepsis [33,34,35,36,37,38,39]. Moreover, the evaluation of I/T2 may enhance the prediction of EOS [37]. These tests are still widely used because they are technically simple and cheap in cost, have a shorter TAT, and do not require advanced laboratory machineries and well-trained laboratory personnel. Unfortunately, most of the studies testing these biomarkers have serious limitations within the design, sample size, and sepsis case definition that limit reliability of results. Moreover, the interpretation of study results is hampered by the evidence that several maternal and neonatal factors, such as maternal blood pressure, gestational age, method of delivery, sex and age in hours of the child, and, finally, the method of blood sampling, can significantly modify all these indices [37]. Similar values have been found in neonates with sepsis, in healthy children, and in subjects with a different disease, making differentiation between infected and non-infected babies practically impossible in most EOS and LOS cases. In a study analyzing complete blood counts from 30,000 healthy neonates, including 852 infants < 28 weeks gestation, ANC measured between birth and the end of the third day of life varied from 1500/mm^3^ to 41,000/mm^3^ and from the 3rd day until the 10th day of life from 1100/mm^3^ to 15,300/mm^3^ [38]. The analysis of data collected in a cohort of 166,092 neonates with suspected EOS and blood cultures revealed that, although low WBC count (<100/mm^3^ and <5000/mm^3^), low ANC (<100/mm^3^), and low I/T (<0.20) were highly specific because they were associated with increasing odds of infection (5.38, 6.84, and 7.97, respectively), all these markers had very poor sensitivity [38]. Generally, it was <20% for all the markers. Only I/T < 20 had a better, although suboptimal, sensitivity, varying from 65.1% to 73.7% according to GA. Moreover, 60% of children with a positive culture had a WBC count in the normal range. Similar findings confirming high specificity and very low sensitivity of these biomarkers for sepsis identification were reported in a study involving neonates with LOS documented by blood cultures [38]. Some advances can be made by deferring determinations until at least 4 h of age in order to reduce the interference of perinatal factors or evaluating serial determinations and categorizing the results into intervals, rather than simply dichotomizing them into normal and abnormal ranges. Although repeated blood drawing cannot be recommended in neonates, Murphy and Weiner demonstrated that two normal I/T ratios correlated with a sterile blood culture and had a maximum negative predictive value of 100%, allowing at least to exclude sepsis even if they could not confirm the diagnosis [39]. Combining these biomarkers with each other or with other biomarkers can improve results, but always with great interpretative limits [40]. On the other hand, interpretation for neutrophils and band forms from stained blood smears is, per se, a limit, as it can significantly vary from laboratory to laboratory [41].

### 3.2. Inflammatory Biomarkers

#### 3.2.1. C-Reactive Protein

C-reactive protein (CRP) is a pentameric acute-phase protein primarily produced by the liver as a response to the insult of various agents. Together with the WBCs and the differential count, CRP has been for years the most used biomarker to identify neonates with sepsis and still remains one of the most common tests in this regard. CRP production is stimulated by proinflammatory cytokines like interleukin (IL)-6, IL-1, and tumor necrosis factor α (TNFα) [28]. The main receptor of CRP is phosphocholine, one of the major components of bacterial membranes. After binding with the receptor, CRP activates the complement cascade favoring phagocytosis and the expression of proinflammatory mediators [42]. The highest levels of CRP are found in serum, and a bacterial infection can cause its values to increase up to 1.000-fold [43]. The CRP serum level begins to rise between 10 and 12 h after the infection onset and peaks at 48 h. When the stimulus ends, CRP values decrease exponentially over 18–20 hours, close to the half-life of CRP [44]. A great number of studies have tested CRP, alone or in combination with other biomarkers, in neonates with EOS or LOS (Table 1) [45,46,47,48,49,50,51,52,53,54,55,56,57,58,59,60,61,62,63,64,65,66,67,68,69,70,71,72,73,74,75,76,77,78,79,80,81,82,83,84,85,86,87,88,89,90,91,92,93,94,95,96,97,98,99,100,101,102,103,104,105,106,107,108,109,110,111,112,113,114,115,116,117,118].

Most of them have relevant methodological problems, have enrolled very few subjects, and have used different definitions of sepsis and different cut-off levels. This explains why results are conflicting and why, together with studies showing adequate sensitivity and/or specificity, several studies report very poor accuracy of CRP for early sepsis identification. Firm conclusions cannot be drawn, although the delayed rise of CRP as a response to infection seems to suggest that a single determination of this protein has an unacceptably low sensitivity for routine use in clinical practice, particularly when EOS is considered. This conclusion is further supported by the evidence that CRP concentrations are significantly influenced not only by infections, but by several other factors also, making the definition of a reliable cut-off value very difficult. CRP spontaneously increases during the first three days of life in a great number of healthy neonates or in babies with non-infective conditions, like a stressful delivery, prolonged labor, meconium aspiration syndrome, delayed transition after birth, prolonged rupture of membranes, hemolysis, intraventricular hemorrhage, or perinatal asphyxias [45,47,116]. Perrone et al. showed that CRP mean values in healthy children were significantly higher at 48 h of life (4.10 mg/L) than at 24 (2.30 mg/L) and 12 h (0.80 mg/L), and that children born by vaginal delivery and emergency caesarean section had a CRP higher than in those born by elective caesarean section (3.80 mg/L and 3.60 mg/L vs. 2.10 mg/L) [117]. Moreover, babies born to a mother that had received, completed or not completed, intrapartum antibiotic prophylaxis had lower CRP values than those born to untreated women (2.90 mg/L and 3.80 mg/L vs. 4.70 mg/L). Furthermore, gestational age (GA) plays a role in conditioning CRP normal levels. Preterm infants have lower CRP levels than term babies with values that were found to increase by 0.405 mg/L for every one week of GA increase. To overcome these limitations, it has been proposed to use CRP with different cut-off levels according to GA and mode of delivery [117], and to perform serial determination within 24–48 h from infection onset in order to evidence CRP progressive increases in neonates developing sepsis [118]. The sensitivity of CRP for the diagnosis of culture-proven EOS increased from 35% to 79% and 89% when serial blood samples were drawn at the initial sepsis workup, after 8–24 h, and after 8–48 h [47].

Despite these limits as a diagnostic marker of sepsis, CRP can be used to exclude sepsis. Normal CRP values in serial controls within a few days from symptom onset are considered indicative of the absence of a bacterial infection [119]. Moreover, CRP can be used to monitor response to antibiotic administration and to decide when antimicrobial treatment can be suspended. Finally, this marker can be used in association with other sepsis markers to improve the accuracy of the diagnosis of both EOS and LOS. Several studies in which CRP values were combined with early sensitive markers such as PCT, IL-6, IL-8, and CD64 have shown an increase in sensitivity between 90% and 100% [120].

#### 3.2.2. Procalcitonin

Procalcitonin (PCT) is a peptide precursor of calcitonin without hormonal activity produced by the liver and, at a lower extent, by monocytes. In healthy individuals outside the neonatal period, serum PCT concentration is extremely low (0.01 µg/L). However, after exposure to pro-inflammatory stimuli, especially of bacterial origin like endotoxins, concentration rises quickly, within 2 to 4 h, peaks within 6 to 8 h, and remains elevated up to 48 h after stimuli are withdrawn [121]. Starting from this evidence, PCT is considered an early-to-intermediate rising biomarker. Synthesis is encouraged by the same cytokines which stimulate CRP production, such as IL-6, IL-1β, and TNF-α, although PCT can also be directly stimulated by bacterial lipopolysaccharides. Contrariwise, PCT is down regulated by interferon-γ, which is commonly produced in response to viral infections [122,123,124]. This explains why PCT is considered a good marker of bacterial infection and a measure to differentiate bacterial from viral infections. Unfortunately, as CRP, in healthy neonates, PCT spontaneously increases after birth, reaches peak values at about 24 h of age, and then decreases gradually by 48–72 h, although with differences according to GA [125,126]. Preterm neonates have an earlier, higher, and longer PCT response than term neonates, showing an inverse relationship between GA and the intensity of neonatal PCT response. Reference PCT values according to GA and days of life have been prepared and used to calculate specific cut-off values for EOS diagnosis [127]. However, their use in this regard is significantly impaired by the evidence that not only bacterial infections, but also several non-infective perinatal circumstances, such as intraventricular hemorrhage, perinatal asphyxia, respiratory distress, hemodynamic instability, and fetal distress, may also raise serum levels of PCT concentrations, making final evaluation very difficult or totally impossible [128,129]. On the contrary, PCT can offer more reliable information for LOS diagnosis as, in children with this condition, physiological variations of PCT serum levels no longer interfere and detected PCT concentrations indicate more precisely the existence of a bacterial infection. Data collected in both preterm and term neonates have shown (Table 2) that sensitivity and specificity values for LOS diagnosis can be even greater than 80%, although with differences according to the cut-off value used to define LOS cases [49,50,51,52,53,54,55,56,57,58,59,60,61,62,63,64,65,66,67,68,69,70,71,72,73,74,75,76,77,78,79,80,81,82,83,84,85,86,87,88,89,90,91,92,93,94,95,96,97,98,99,100,101,102,103,104,105,106,107,108,109,110,111,112,113,114,115,116,117,118,119,120,121,122,123,124,125,126,127,128,129,130,131,132,133,134,135].

Values > 2 µg/L were those associated with the highest sensitivity and specificity, whereas lower cut-off values were less effective in identifying children with LOS [54]. However, as for CRP, the accuracy of PCT in EOS and LOS diagnosis increases significantly when serial determinations within a few hours are performed. Persistently low PCT levels exclude EOS and LOS. Moreover, in positive cases, normalization of PCT concentrations can be used to decide the discontinuation of antibiotic therapy [136]. Finally, the combined use of PCT and other laboratory markers can improve information [53].

#### 3.2.3. Serum Amyloid A

Similar to CRP and PCT, serum amyloid A (SAA) is an acute phase reactant. It is synthetized in the liver and, to a lower extent, in smooth muscle cells, macrophages, adipocytes, and endothelial cells in response to several stimuli, including infections [137]. Its production occurs under IL-1, IL-6, TNF-α, and Gram-negative bacteria lipopolysaccharides (LPBs) stimulation with concentrations that significantly vary according to age [28]. The lowest levels are seen in cord blood while the highest levels were observed in elderly patients [138]. The kinetic characteristics of SAA seem to suggest that it could be a reliable biomarker of neonatal sepsis, as it increases within a few hours from sepsis onset and returns to baseline levels after four days. Compared with CRP, SAA levels rise earlier and sharper, reach higher levels, and return faster to normal values when infection is cured [67]. Moreover, in a study in which SAA was compared to several other biomarkers of neonatal sepsis, it was found to be the most favorable and promising marker for diagnosis and monitoring of response to treatment [139]. The efficacy of SAA for early diagnosis of both EOS and LOS was confirmed by most of the studies testing SAA in clinical practice [140,141]. Superiority over CRP was reported by Arnon et al. [61]. These authors showed that serum SAA measured at disease onset had better accuracy for predicting EOS than CRP (sensitivity 96% vs. 30%, specificity 95% vs. 98%, positive predictive value 85% vs. 78%, negative predictive value 99% vs. 83%). However, this finding was not confirmed in the meta-analysis by Yuan et al. in which 9 studies enrolling 823 preterm and term neonates with EOS and LOS were evaluated [142]. Pooled sensitivity and specificity of the SAA test for the diagnosis of neonatal sepsis at disease onset were 84% and 89%, respectively. Only slightly lower values were calculated 8–96 h after the first suspicion of sepsis with a pooled sensitivity of 78% and specificity of 84%. The sensitivity and specificity of CRP were substantially similar. The heterogeneity of the studied population and difference in cut-off values used to define normal and abnormal values of both biomarkers may explain these differences. On the other hand, it cannot be forgotten that SAA, like CRP and PCT, rises up in response to non-infective stimuli, for example, stressful delivery and intraventricular hemorrhage, and that the role of GA in conditioning SAA levels is not definitively established.

#### 3.2.4. Proadrenomedullin

Adrenomedullin is a peptide produced by heart, adrenal medulla, lungs, kidneys, and vascular endothelium during physiological stress. It regulates the vascular tone, favoring organ perfusion, and exerts a significant antibacterial and immunomodulatory response [143]. A precursor of adrenomedullin, proadrenomedullin (ProADM), has been tested as biomarker of severe bacterial disease in both children and adults. It was shown that ProADM sharply increases shortly after infection and is a good indicator of disease severity and death risk. Data collected in neonates seem to suggest that ProADM can be used to diagnose EOS and to predict response to antibiotic therapy. In a study enrolling 60 newborn infants with sepsis proven with positive blood cultures and 30 healthy neonates, pro-ADM serum concentrations were significantly higher (14.39 ± 0.75 nmol/L) in the sepsis group than in controls (3.12 ± 0.23 nmol/L). Sensitivity for the diagnosis of sepsis was 93.3%, and specificity 86.7% [144]. However, as ProADM serum values are higher in preterm than in term babies, better prediction of EOS depends on the use of different cut-off levels according to GA (3.9 nmol/L in term neonates and 4.3 nmol/L in preterm babies) [96,145]. Better results have been reported when ProADM was used in combination with other markers [146].

#### 3.2.5. Other Inflammatory Markers

Adipokines such as visfatin and resistin, hepcidin, progranulin, stromal cell-derived factor1, endocan, and pentraxin-3 play a role in immune system response and inflammation development and have been indicated as potential markers of sepsis in neonates [77,147,148,149,150,151]. However, studies in this regard are very few and further information is needed to draw firm conclusions.

#### 3.2.6. Cytokines

After infecting pathogens are recognized by toll-like receptors, host immune response is initiated mainly by the release of proinflammatory cytokines from macrophages and monocytes [91,152]. Because of this early involvement in the host immune response to infections, cytokines have been considered as promising biomarkers of neonatal sepsis, especially in recent years when most problems of cytokine detection in blood samples have been solved [153]. Moreover, as CRP and PCT production depends on cytokine release, it was thought that the measure of cytokines could offer an earlier and more effective evaluation of sepsis development compared to the traditionally used biomarkers. Unfortunately, not all the expected benefits have materialized.

##### Interleukin 6

IL-6 is released within 2 h after the onset of bacteremia, peaks at approximately 6 h, and declines over the following 24 h. Moreover, it can be detected in the blood of neonates 1–2 days before the clinical presentation of culture-proven sepsis [154]. Finally, when septic patients receive appropriate antibiotic treatment, IL-6 decreases precipitously back to the baseline non-infectious state within 24 h [155]. These characteristics greatly limit the role of IL-6 as clinically useful biomarkers across all EOS and LOS phases, including the monitoring of treatment efficacy and duration. Moreover, the potential use of IL-6 for early identification of infected neonates at risk of EOS development is hampered by the evidence that this cytokine is an important mediator of host response to stress and tissue injury [17] and increases even in uninfected neonates with hypoxia, fetal distress, prematurity, chorioamnionitis, mechanical ventilation, surfactant therapy, meconium aspiration, and intrauterine growth retardation [88,156]. Table 3 summarizes the main studies on IL-6 for the diagnosis of neonatal sepsis [157,158,159,160,161,162,163,164,165,166,167,168,169,170,171,172,173,174,175,176,177].

Despite cut-off limits for this marker not being definitively established, the serial measurement of IL-6 or combinations with other specific biomarkers of infection could improve the diagnostic potential of IL-6. Berka et al. assessed IL-6 at 2 h and at 12–24 h after delivery in very preterm neonates and found that increase of IL-6 values to >200 ng/L could diagnose EOS with a sensitivity of 89% and specificity of 77% [178]. The negative predictive value was 98%. The same authors in a retrospective case-control study identified values of IL-6 < 100 ng/L e CRP < 10 mg/L as accurate cut-offs for ruling out LOS at clinical onset [179]. Finally, recent studies have shown that IL-6 could be used to define the etiology of sepsis. Significantly greater inflammatory response in gram-negative sepsis than in gram-positive sepsis has been demonstrated; Celik et al. observed a cut-off level of 202 pg/mL for IL-6 differentiated gram-negative from gram-positive sepsis with 68% sensitivity and 58% specificity [89]. It has been observed (177) that IL-6 (>400 pg/mL) alone or in combination with TNF-α (>32 pg/mL), IL-8 (>200 pg/mL), and granulocyte-colony stimulating factor (>1000 pg/mL) had 100% sensitivity, specificity, negative predictive value, and 38–69% positive predictive value to differentiate gram-negative neonatal sepsis [180].

##### Interleukin-8

IL-8 has kinetic characteristics very similar to those of IL-6 and, like this, can increase in newborns regardless of the presence of an infection. It therefore has the same limitations, especially for the early diagnosis of EOS. A meta-analysis of eight studies enrolling neonates with documented sepsis reported that IL-8 had a global sensitivity and specificity for sepsis diagnosis of 78% and 84%, respectively [181]. However, definitive conclusions could not be drawn as studies used different cut-off levels and included both EOS and LOS. However, the accuracy of IL-8 seems increased when it is combined with other biomarkers, mainly CRP. In a study enrolling preterm infants, it was shown that, although IL-8 had low sensitivity (48.15%) as a marker of LOS, a combination with CRP increased sensitivity to 78.12% [182].

##### Tumor Necrosis Factor

Tumor necrosis factor (TNF) is a potent pro-inflammatory cytokine with a major role in initiating a cascade of activation of other cytokines and growth factors in inflammatory responses. TNF stimulates IL-6 production and is inhibited by IL-6 itself [183]. TNF levels rise immediately after exposure to an infectious agent, have a peak at about 1 h, and disappear within 3 h [184]. These characteristics explain why attempts to use TNF as an early marker of sepsis have failed. Generally, the determination of cytokines a few hours after infection initiation reveals high IL-6 values, whereas TNF is no longer detectable [185].

### 3.3. Cell Adhesion Molecules

Several cell adhesion molecules presepsin (P-SEP), cluster differentiation molecule-64 (CD64) CD11b, sCD163, soluble trigger receptor expressed on myeloid cell-1 (sTRIM1), and pentraxin3 were tentatively used to differentiate septic neonates from healthy subjects. Only presepsin, CD14, and sTRIM1 were used in a number of studies useful for drawing some conclusion regarding their role in this regard [186].

#### 3.3.1. Presepsin

Presepsin (P-SEP) is the soluble N terminal fragment of CD14, a cell surface glycoprotein expressed by various innate immunity cells, like monocytic and neutrophils. In case of bacterial infection, interaction between CD14 and bacterial components such as LPBs activates a proinflammatory pathway through toll-like receptor 4 (TRL-4) that leads to an internalization of the complex. During this process, CD14 is proteolyzed by cathepsin D, a lysosomal protease, and this results in the releasing of its soluble part, P-SEP, in the circulation [187]. P-SEP kinetic studies have shown that blood concentration of this biomarker starts to increase within 2 h after induction, peaks at 3 h, and remains elevated for up to 4–5 h [188]. From this, it was concluded that P-SEP could be used for an early identification of neonatal sepsis. Two meta-analyses, including studies carried out in neonates with both EOS and LOS, seemed to confirm this assumption [189]. However, most of these studies had significant problems. The role of maternal or child factors, including GA, birth weight, type of delivery, and maternal infections in conditioning P-SEP accuracy was not defined. Moreover, the interference of the physiological variations of P-SEP values in the first days of life were not considered. These limitations have raised doubts about the real role of the P-SEP marker of sepsis in neonates [190]. A recent meta-analysis including 12 studies of preterm or term babies with EOS or EOS and LOS has better defined the relevance of several maternal or neonatal factors in conditioning P-SEP accuracy for neonatal sepsis diagnosis. It was calculated that the accuracy of this marker for an early detection of neonatal sepsis was slightly better in cases of EOS than in cases of LOS. This is because studies enrolling only newborns with EOS showed higher specificity compared with those enrolling a mixed population of EOS and LOS (0.99; 95% CI, 0.80–1.00 vs. 0.89; 95% CI, 0.82–0.93; *p*  =  0.003), but not a significantly different sensitivity (0.96; 95% CI, 0.85–0.99 vs. 0.92; 95% CI, 0.85–0.96; *p*  =  0.35). Finally, P-SEP accuracy was not associated with GA and the method used for marker detection. Moreover, recent studies have led to the definition of P-SEP cut-off values for healthy term and preterm neonates in the first three days of life, favoring early identification of neonates with EOS [191]. Table 4 summarizes the main studies on the accuracy of presepsin for the diagnosis of neonatal sepsis [192,193,194,195,196,197,198,199,200,201].

Starting from these findings, P-SEP is presently considered a promising biomarker for the diagnosis of EOS. Further studies are, however, needed to precisely define cut-off values for the diagnosis of LOS and to monitor response to therapy and sepsis evolution. Finally, the potential use of P-SEP in association with other biomarkers should be better studied. A recent evaluation has shown that the diagnostic efficacy of P-SEP was highest when used in combination with IL-6 and CRP compared when the biomarker was used alone. The area under the Rock curve (AUC) for discriminating the probable infection group from the unlikely infection group was 0.97 (95% CI: 0.911–0.990) vs. 0.845 (95% CI: 0.708–0.921) [202].

#### 3.3.2. Soluble Triggering Receptor

The triggering receptor expressed on myeloid cells-1 promotes the release of proinflammatory cytokines and chemokines [203,204]. Studies carried out in neonates have shown that the soluble form of this compound (sTREM1) increases in serum after exposure to infectious agents, that sTREM-1 levels in neonatal plasma were comparable with those in adults, and that GA, maternal age, birth weight, way of delivery, sex, intrauterine growth restriction, and pre-labor rupture of the membranes do not influence sTREM1 concentrations [205].

Studies in neonates with suspected or documented sepsis have shown that the measure of this marker can differentiate septic neonates from healthy individuals. Adly et al. reported that baseline levels of this marker were significantly higher in septic neonates (*p* < 0.001), although higher in preterm babies and in those with EOS [206]. Moreover, after 48 h of antibiotic treatment, sTREM1 concentrations were significantly lower than at baseline. However, when compared to other sepsis markers, results of the studies were conflicting. Compared to CRP and PCT, sTREM1 was found to have higher sensitivity (82% vs. 45% of CRP and 55% of PCT), but lower specificity (48% vs. 82% of CRP and 75% of PCT) [207]. On the contrary, when compared to IL-6, no advantage of sTREM1 use was evidenced [208].

#### 3.3.3. Cluster Differentiation Molecule-64

Cluster differentiation molecule-64 (CD64) expressed from neutrophils and monocytes facilitates phagocytosis and intracellular killing of opsonized micro-organisms. Its expression increases 5–10 times the normal limit 1–6 h after bacterial infection or inflammatory stimuli [209]. Moreover, its expression is not influenced by GA, maternal, perinatal, or postnatal factors. For this, it was considered a potential useful biomarker of neonatal sepsis. However, results of clinical studies have reported conflicting results due to the large range of sensitivity (26–95%) and specificity (62–97%) in different individual studies [210,211,212,213]. In a meta-analysis of 17 studies including 3478 neonates, the overall pooled sensitivity, specificity, positive likelihood ratio, and negative likelihood ratio were 77% (95% CI 0.74–0.79), 74% (95% CI 0.72–0.75), 3.58 (95% CI: 2.85–4.49), and 0.29 (95% CI: 0.22–0.37), respectively. However, subgroup analysis revealed higher sensitivity and specificity in term infants than those in preterm infants, and the authors concluded that information due to this biomarker should be treated with caution. More accurate results could be obtained combining CD64 with other sepsis biomarkers [214].

## 4. Future Biomarkers: Omics Technologies and Personalized Medicine

Omics technologies have recently been used to identify markers of sepsis in neonates. The information derived in this regard are presently very poor, and it seems premature to think that they are used in daily clinical practice. However, it seems likely that when methods will be standardized and more information will be available, they will have a prominent diagnostic place. Some examples may suggest what information can be obtained and how it allows to individualize the diagnostic and therapeutic process much more than is possible with traditional markers.

Recently developed molecular biology methods such as microarrays and next-generation sequencing technologies have allowed to simultaneously evaluate expression changes of thousands of genes at the cellular level at the onset of sepsis and during it. Using microarray, Smith et al. identified a 52-gene network including genes from innate and adaptive immunity that could distinguish neonates with bacterial infections from healthy controls with 98% accuracy [215]. Similar findings were reported by Cernada et al. [216]. Gene expression analysis evidenced that 554 genes mainly linked to cytokine secretion could discriminate VLBW neonates with sepsis from controls with 100% sensitivity and 68% specificity.

Important information on neonatal sepsis can also be derived from the evaluation of gene expression mediated by epigenetic mechanisms. As microRNAs (miRNAs) can significantly influence posttranscriptional regulation of gene expression playing an important role in the development of immune system functions, inflammatory response, and sepsis development [217,218], the use of miRNAs as potential markers of sepsis was considered. Initial studies in this regard have shown that in adult patients with sepsis, serum concentrations of several miRNAs could be associated with the risk of disease development, and in patients with disease, could anticipate prognosis [219]. Studies in neonates are few, but some of them have clearly evidenced that miR-16, miR-16A, nmiR96-5p, miR-141, miR-181.a, and MIR-1184 have substantial diagnostic potential for neonatal sepsis monitoring [184]. Levels of different miRNAs in babies with sepsis are higher or lower than in healthy matched children, according to the role played by the single miRNA in the immune system function. In general, overexpression of miRNA is associated with increased concentrations of pro-inflammatory cytokines, and the opposite occurs when a miRNA downregulates inflammatory markers [185].

Metabolomic phenotyping of septic neonates using nuclear magnetic resonance imaging (NMR) and mass spectrometry can also be used for the diagnosis of neonatal sepsis.

Volatile organic compound (VOC) analysis on various sample types through different techniques is a non-invasive method to monitor modifications of cellular metabolism and gut microbiota composition. Several studies have shown that VOC originated from the gut are different in healthy subjects than in those with certain diseases or risk conditions, and that VOC analysis can lead to an early and accurate detection of inflammatory bowel diseases, cancer, Alzheimer’s disease, and preterm birth. Recently, fecal VOC profiles of neonates were studied with various recognition techniques. The importance of an early VOC analysis was evidenced, as it can allow for preclinical discrimination between infants developing LOS and matched controls. Berkhout et al. compared VOC profiles of 127 preterm infants with LOS to those of 127 matched healthy controls at 3, 2, and 1 day before clinical LOS onset [220]. Deep differences between groups at all three predefined time points were evidenced, regardless of LOS etiology, although the highest accuracy rates were obtained for infections due to Escherichia coli, followed by Staphylococcus aureus and Staphylococcus epidermidis. Conclusions were that VOS analysis could have a high predictive value up to 3 days before the clinical onset of LOS.

More recently, a well conducted study has confirmed the potential of VOC as an early, non-invasive biomarker for LOS, allowing to deepen the role of the methods used to detect VOCs and the etiology of LOS in conditioning the discriminatory capacity of the test [221]. Data collected in 121 LOS preterm infants and 121 matched controls have indicated that the use of gas chromatography-ion mobility spectrometry (GC-IMS) for feces analysis offers better results than gas chromatography coupled to time-of-flight mass spectrometry (GC-TOF-MS). With GC-IMS, differences between LOS cases and healthy babies can be detected already 3 days before LOS onset, whereas GC-TOF-MS analysis can reveal differences only significantly closer to disease development. Moreover, identification of babies at risk of LOS occurs earlier when gram-negative rods are the disease agents. Finally, differences according to single agents were identified. In cases due to Staphylococcus aureus, VOCs were discriminative from controls at three days before LOS. On the contrary, when coagulase negative strains were the infecting agents, discrimination was possible only when all time points were combined. Despite these interesting results, it seems mandatory that before VOC analysis can enter in routine clinical practices, further studies are needed. The methods used to detect VOCs are expensive, time-consuming, and require highly trained operators. Only simplified tests can have a future in neonatal sepsis diagnosis. On the other hand, no data have been collected in term neonates and in children with EOS, and no information is available regarding the role of previous prophylactic antibiotic therapy, frequently given in neonates before LOS development in conditioning VOC analysis results.

## 5. Conclusions

Blood cultures, still considered the gold standard for neonatal sepsis diagnosis, have several limitations, mainly the very low sensitivity and the long TAT, that preclude its routine use as sole marker of neonatal sepsis in clinical practice. To overcome this problem, in the last thirty years, several efforts to find more reliable alternatives have been made. Unfortunately, none of the markers that have been proposed fulfills all the criteria for becoming an ideal marker. White blood cell count and differential count have very low accuracy in identifying neonates with sepsis and allow, at most, to exclude the disease. Acute phase reactants, including CRP and PCT, are the most used markers. Both have several limitations. They, particularly CRP, have non-ideal kinetic characteristics and are strongly influenced by pre-, peri-, and postnatal factors, making it very difficult to establish specific cut-off levels. Some advantage may perhaps be offered by SAA, even if for this marker, reliable and definitive data on the role of some pre- and postnatal factors in influencing serum levels are lacking and effective cut-off levels are not definitively established. Similar conclusions can be drawn when the results of studies regarding cytokine use are considered. The study of the immune system response to infections has led to the identification of some markers, including cell-adhesion molecules, potentially useful in the identification of neonates with sepsis. Presepsin is the one more largely studied, but for this biomarker also, available data are not enough to suggest its routine use in clinical practice. The application of omics technologies to the diagnosis and treatment of neonatal sepsis could lead to the identification of novel biomarkers. Studying sepsis across the transcriptional and metabolic response at different times can allow us to understand interactions between genes and biomolecules, and to identify not only children at risk or with defined disease, but those with the most complicated course. A personalized intervention would be possible. Unfortunately, these technologies are still in development and several years will have to pass before they can be routinely used in the NICU.

In conclusion, despite intense research, the diagnosis of neonatal sepsis and the conduct of antibiotic therapy cannot be at present decided on the basis of a single biomarker. Given the importance of the problem and the need to reduce the abuse of antibiotics, further studies are urgently required. However, instead of looking for new biomarkers, it seems easier and more productive to test combinations of two or more of the presently available biomarkers. Combining results of cytokine and traditional inflammatory markers determination may be a potential solution, especially when serial measurements are performed. Moreover, studies based on omics technologies should be strongly boosted. However, while waiting for new information, the use of the clinical scores prepared by some scientific institutions could be suggested. Based on maternal risk factors and infant clinical indicators, sepsis risk can be calculated and a significant reduction of antibiotic consumption can be obtained.

## Figures and Tables

**Table 1:** Main studies on C-reactive protein (CRP) accuracy for the diagnosis of neonatal sepsis.

Reference	Population	EOS or LOS	Cut-off Value	Sensitivity	Specificity	PPV	NPV
Sharma 1993 [45]	Full-term and preterm: Group A (10 proved sepsis group) vs. Group B (24 probable sepsis) vs. Group C (16 no sepsis)	EOS and LOS	6 mg/L	80%	93.8%	ND	ND
Ng 1997 [46]	Preterm 35 infected vs. 46 non-infected vs. 20 controls	LOS	12 mg/L	84%	96%	95%	87%
Benitz 1998 [47]	Full-term and preterm: Proven sepsis 20 vs. Probable sepsis 74 vs. No sepsis 908	EOS	1 mg/dL	35%	90%	6.7%	98.6%
		LOS		61.5%	68.9%	43.8%	82%
Doellner 1998 [48]	Full-term and preterm 24 Group 1 (infection) vs. 18 Group 2 (probable infection) vs. 31 Group 3 (mixed group) vs. 94 Group 4 (negative sepsis) vs. 70 Group 5 (control)	EOS and LOS	10 mg/L	96%	74%	49%	99%
Enguix 2001 [49]	Mixed population: 20 septic neonates vs. 26 controls	LOS	23 mg/L	95.8%	83.6%	80.2%	96.7%
Manucha 2002 [50]	Full-term and preterm: 21 proved sepsis vs. 129 probable sepsis vs. 40 no sepsis	EOS	6 mg/L	76%	79%	37%	96%
Blommendahl 2002 [51]	Full term and preterm 219	ND	1 mg/L	58%	84%	24%	94%
Guibourdenche 2002 [53]	Full term and preterm: 88 non-infected; 21 infected; 10 unclassified	EOS	7.5 mg/L	68%	80%	81%	72%
Chiesa 2003 [17]	134 consecutives critically ill newborns: 19 cases and 115 controls	EOS	At birth 4 mg/L	73%	83%	ND	ND
			At 24 h 10 mg/L	91%	87%		
			At 48 h 10 mg/L	91%	84%		
Santana Reyes 2003 [53]	Full-term and preterm: Group 1 (20 infected) vs. Group 2 (20 noninfected) vs. Group 3 (20 control)	EOS and LOS	ND	80%	92%	ND	ND
Vazzalwar 2005 [54]	Preterm: 36 infected, 15 non-infected, 16 controls	LOS	0.8 mg/dL	72%	93%	96%	58%
Arnon 2005 [55]	Preterm 23 proven sepsis; 15 clinical sepsis; 78 controls	LOS	10 mcg/mL	32%	97%	86%	74%
Verboon-Maciolek 2006 [56]	Mixed population 111 patients	LOS	14 mg/L	65%	52%	63%	54%
Turner 2006 [57]	33 preterm infants	LOS	10 mg/L	74%	39%	46%	68%
			20 mg/L	47%	89%	75%	70%
			30 mg/L	41%	96%	87%	69%
			50 mg/L	31%	98%	91%	67%
Resch 2007 [58]	16 proven sepsis, 25 clinical sepsis, 8 uncertain, 27 non-infected	EOS	2.5 mg/L	69%	96%	96%	67%
			8 mg/L	49%	100%	100%	58%
Arnon 2007 [59]	Full-term 23 cases vs. 71 controls	EOS	7 mg/L	30%	98%	78%	83%
Ucar 2008 [60]	Full term and preterm: 36 cases vs. 36 controls	LOS	0.8 mg/dL	Day 0: 97.2%	100%	ND	ND
				Day 4: 100%	100%		
				Day 8: 100%	100%		
Fendler 2008 [61]	78 preterm newborns	LOS	0.22 mg/dL	85%	88.9%	97.1%	57.1%
Schrama 2008 [62]	Full-term and preterm: Documented sepsis (24) vs. Suspected sepsis (77) vs. Control (55)	EOS and LOS (Sepsis vs. control)	10 mg/L	92%	99%	ND ND	
		EOS and LOS (Sepsis vs. suspected infection and control)		92%	85%		
		EOS and LOS (Sepsis and suspected infection vs. control)		80%	67%		
Boo 2008 [63]	Full term and preterm: 87, 18 with confirmed sepsis	EOS and LOS	ND	55.6%	89.9%	ND	ND
Sherwin 2008 [64]	Full-term and preterm Group 1 (culture positive) vs. Group 2 (culture-negative)	EOS and LOS	38 pg/mL	22%	92%	31%	88%
Jacquot 2009 [65]	Preterm: 30 cases vs. 43 controls	LOS	10 mg/L	58%	86%	74%	75%
Zaki 2009 [66]	Full-term and preterm: Group 1 (58 infected) vs. Group 2 (32 noninfected) vs. Group 3 (30 control)	EOS and LOS	8 mg/L	86%	97%	96%	88%
Çetinkaya 2009 [67]	Preterm: Group 1 (highly probable sepsis) vs. Group 2 (probable sepsis) vs. Group 3 (possible sepsis) vs. Group 4 (no sepsis)	EOS and LOS	0.5 mg/dL	72.3%	100%	100%	54%
Groselj-Grenc 2009 [68]	17 Neonates with SIRS vs. 29 controls	LOS	11 mg/L	59%	100%	100%	89%
Rego 2010 [69]	144 preterms presenting respiratory distress: 44 infected, 100 uninfected	EOS	0.6 mg/dL	76%	70%	52%	87%
Celik 2010 [70]	Full-term and preterm: Group 1 (170 clinical and proven sepsis) vs. Group 2 (62 noninfected)	EOS and LOS	5.82 mg/L	71%	97%	99%	49%
Edgar 2010 [71]	Full-term and preterm 74 Infected; 118 Non-infected; 27 Controls	EOS	0.6 mg/L	61.5%	82.3%	36.3%	92.8%
		LOS	0.4 mg/L	71.2%	55.6%	71.2%	55.6%
Kumar 2010 [72]	Full-term and preterm 83 Proven sepsis vs. 94 probable sepsis	EOS and LOS	5 mg/dL	95.2%	85.3%	80.6%	96.5%
				98.9%	83.3%	80.9%	99.1%
Hotoura 2011 [73]	Full-term Group 1 (20 suspected infection) vs. Group 2 (25 sepsis) vs. Group 3 (50 infection-free control subjects)	EOS and LOS	10 mg/L	64%	78%	60%	81%
Campolat 2011 [74]	74 preterm infants with history of pPROM: 32 infected, 42 uninfected	EOS	0.72 mg/dL	56%	58%	ND	ND
Altunhan 2011 [75]	Full term and preterm: Group 1: 171 suspected sepsis vs. Group 2: 89 control group	EOS	At birth 5 mg/L	44.5%	59.4%	45.6%	64.2%
			At 24 h of life 12 mg/L	76.4%	78.9%	79.7%	81.6%
Naher 2011 [76]	Full-term and preterm Group 1 (highly probable sepsis); Group 2 (probable sepsis); Group 3 (possible sepsis); Group 4 (no sepsis)	EOS and LOS	6 mg/L	55%	100%	100%	35.7%
Cekmez 2011 [77]	Full term or near term (>34 wks): 62 cases vs. 43 controls	LOS	0.82 mg/dL	82%	79%	ND	ND
Bohnhorst 2012 [78]	Preterm Proven infection (58) vs. Unproven infection (112)	LOS	10 mg/L	69%	84%	69%	84%
Adib 2012 [79]	Full term and preterm: 20 confirmed sepsis vs. 49 clinical sepsis vs. 18 controls	EOS and LOS	12 mg/L	45%	95%	30%	30%
Choo 2012 [80]	Full-term and preterm Group 1 (11 documented sepsis): Group 2 (12 clinical sepsis): Group 3 (14 control)	EOS and LOS	10 mg/L	9%	83%	33%	50%
Adollahi 2012 [81]	Full term and preterm: 30 proven EOS; 19 clinical EOS; 16 negative infectious status; 30 uncertain infectious status	EOS	2.5 mg/L	69%	96%	96%	67%
			8 mg/L	49%	100%	100%	58%
Ertuğrul 2013 [82]	Premature	LOS	ND	58.3%	80%	77.8%	61.5%
Park 2014 [83]	Full term and preterm: 18 confirmed sepsis, 56 suspected sepsis, 81 mild infection, 114 controls.	ND	6 mg/L	100%	78.1%	24.7%	100%
			10 mg/L	100%	85.7%	33.3%	100%
Steinberger 2014 [16]	Preterm infants with risk factors for EOS: 30 infected, 188 uninfected	EOS	0.55 mg/L	56.3%	93.5%	56.3%	93.5%
			8.00 mg/L	12.5%	99.1%	66.7%	88.4%
Hisamuddin 2015 [84]	Full-term and preterm Group 1 (43 confirmed sepsis); Group 2 (104 no sepsis)	EOS and LOS	5 mg/dL	76.92%	53.49%	80%	48.94%
Decembrino 2015 [85]	Full-term and preterm Group 1 (8 sepsis); Group2 (33 suspected sepsis)	EOS and LOS	6 mg/L	50%	66.7%	ND	ND
Kipfmueller 2015 [86]	Preterm 7 confirmed sepsis; 10 clinical sepsis; 8 indeterminate	LOS	10 mg/L	43%	83%	ND	ND
Pynn 2015 [87]	Full-term and preterm 37 culture positive sepsis vs. 102 negative evaluations	LOS	10 mg/L	82%	66%	50%	90%
Al-Zaharani 2015 [88]	Full-term and preterm: 34 proven EOS, 37 suspected EOS, 29 no EOS	EOS	2.5 mg/L	91.1%	72.4%	94.2%	77.7%
Çelik 2015 [89]	Full-term and preterm: 40 proven sepsis, 76 clinical sepsis, 111 control	EOS and LOS	0.16 mg/dL	75%	76.3%	50.8%	91.9%
Abdel Mohsen 2015 [90]	Full-term and preterm: 35 cases vs. 35 controls	EOS	12 mg/L	72.9%	100%	93.2%	69.7%
Yang 2016 [91]	Full term and preterm: 60 cases and 60 controls	LOS	4.07 mg/L	38.6%	95.1%	89.4%	59.1%
Ganesan 2016 [92]	Full-term and preterm Group 1 (40 suspected cases); Group 2 (40 control)	EOS and LOS	13.49 mg/L	80%	65.7%	25%	95.83%
Sabry 2016 [93]	Mixed, term and preterm: 80 cases vs. 40 controls	EOS and LOS	2.65 mg/L	82.5%	77.5%	88%	68.9%
Tabl 2016 [94]	Full-term: 22 cases, 28 non-infectious SIRS, 20 healthy controls	EOS and LOS	ND	81.8%	64.6%	51.4%	88.6%
Ozdemir 2016 [95]	Full-term: 29 EOS vs. 40 controls	EOS	6.35 mg/L	83%	75%	97%	75%
Abd Elmouttaleb 2016 [96]	Gestational age 36–40 wks: 50 cases vs. 30 controls	EOS	6 mg/dL	51.6%	70.7%	40.5%	78.2%
Ahmed 2017 [97]	Full term and preterm 135 newborns	EOS and LOS	5 mg/dL	98.03%	91%	97%	93.7%
He 2017 [98]	Preterm (>34 wks) and term infants with suspected EOS: 68 infected, 83 uninfected	EOS	3 mg/L	67.65%	66.27%	62.16%	71.43%
Chen 2017 [99]	Mixed, term and preterm: 96 EOS vs. 44 Non-infective SIRS vs. 53 healthy controls	EOS	9.9 mg/L	77.1%	88.6%	ND	ND
Montaldo 2017 [100]	Preterm (<34 wks gestational age): 32 cases vs. 38 controls	EOS	4.3 mg/L	42%	82%	82%	45%
Beltempo 2018 [101]	416 VLBW	EOS	10 mg/L	49%	76%	43%	79%
Utkarshni 2018 [102]	Mixed population, full term and preterm (50)	LOS	6 mg/L	66.6%	73.1%	35.2%	ND
Rashwan 2019 [103]	Full-term and preterm Group 1 (102 proven sepsis); Group 2 (66 probable sepsis)	EOS and LOS	6 mg/dL	79.4%	93.3%	96.4%	66.7%
Kumar 2019 [104]	Mixed, term and preterm: 41 cases vs. 41 controls	EOS	3.2 mg/dL	75%	97.5%	91.6%	82.6%
		LOS		88.2%			
Khan 2019 [105]	Full-term and preterm 269 EOS 116 LOS	EOS	5 mg/dL	17.2%	58.3%	72.3%	9.8%
Wu 2019 [106]	Full-term and preterm Sepsis (195) vs. Control (100)	EOS and LOS	47.33 mg/L	71%	75.38%	ND	ND
Ahmed 2019 [107]	Mixed, term and preterm (birth weight more than 1500 gr): 30 cases vs. 30 controls	EOS	1.5 mg/dL	66.7%	73.8%	52.2%	83.8%
Stoicescu 2019 [108]	Mixed, term and preterm: 37 cases vs. 49 controls	EOS and LOS	All patients: 0.45 mg/dL	73.5%	68.4%	69.4%	74.3%
			EOS: 0.45 mg/dL	70.4%	66.7%	63.3%	75%
			LOS: 0.65 mg/dL	75%	88.9%	60%	94%
Değirmencioğlu 2019 [109]	Preterm (≤32 wks of GA): 26 cases vs. 29 controls	LOS	3.9 mg/L	81.5%	72.2%	73.6%	81.4%
El-Madbouly 2019 [110]	Full-term: 30 cases vs. 30 controls	EOS and LOS	6 mg/L	85.2%	39.0%	67.6%	64.0%
Khater 2020 [111]	Mixed, term and preterm: 40 proved sepsis vs. 50 suspected sepsis vs. 30 controls	EOS and LOS	9 mg/mL	72%	61%	29%	82%
Hashem 2020 [112]	Mixed, term and preterm: 133 cases vs. 102 controls	EOS and LOS	6 mg/L	71.0%	94.1%	93.9%	71.6%
Morad 2020 [113]	Full term and preterm: 50 neonates with clinically suspected sepsis (31 positive culture)	EOS and LOS	10 mg/dL	89.5%	66.7%	92.5%	60%
Yang 2020 [114]	152 preterm (>34 wks) and term infants a risk for EOS: 76 infected, 76 uninfected	EOS	3.5 mg/L	73.7%	57.9%	63.3%	69.4%
Tang 2022 [115]	Full term and preterm 169	EOS and LOS	15 mg/L	75%	84%	14%	99%

Table Legend: EOS (early onset sepsis); LOS (late onset sepsis); PPV (positive predictive value); NPV (negative predictive value); wks (weeks); GA (gestational age); SIRS (systemic inflammatory response syndrome); ND (not declared); pPROM (Preterm Premature Rupture of Membranes).

**Table 2:** Main studies on procalcitonin (PCT) accuracy for diagnosis of neonatal sepsis.

Reference	Population	EOS or LOS	Cut-off Value	Sensitivity	Specificity	PPV	NPV
Enguix 2001 [49]	Mixed population: 20 septic neonates vs. 26 controls	LOS	6.1 ng/mL	98.6%	88.9%	89.5%	98.5%
Blommendahl 2002 [51]	Full term and preterm 219	ND	1 mg/mL	77%	62%	16%	97%
Guibourdenche 2002 [53]	Full term and preterm: 88 non-infected; 21 infected; 10 unclassified	EOS	2.5 mg/L	87%	90%	86%	93%
Chiesa 2003 [17]	134 consecutives critically ill inborns: 19 cases and 115 controls	EOS	At birth 1 μg/L	82%	95%	ND	ND
			At 24 h 100 μg/L	100%	96%		
			At 48 h 50 μg/L	91%	100%		
Vazzalwar 2005 [54]	Preterm: 36 infected, 15 non-infected, 16 controls	LOS	0.5 ng/mL	94%	36%	45%	92%
			1.0 ng/mL	78%	64%	54%	84%
Verboon-Maciolek 2006 [56]	Mixed population 111 patients	LOS	0.5 mcg/L	69%	82%	83%	68%
Turner 2006 [57]	33 preterm infants	LOS	0.5 ng/mL	74%	54%	53%	78%
			1 ng/mL	48%	88%	73%	73%
			2.3 ng/mL	48%	97%	91%	74%
Resch 2007 [58]	16 proven sepsis, 25 clinical sepsis, 8 uncertain, 27 non-infected	EOS	6 ng/mL	77%	91%	93%	72%
			2 ng/mL	83%	61%	76%	70%
			14 ng/mL	63%	100%	92%	64%
López Sastre 2007 [135]	Full term and preterm: 31 confirmed vertical sepsis vs. 38 vertical clinical sepsis vs. 79 non-infectious disease vs. 169 asymptomatic	EOS	0.15 ng/mL, at birth	91.2%	91.8%	28.%	76.2%
			1.2 ng/mL, 12–24 h of life	90.2%	43%	39%	91.5%
			0.75 ng/mL, 26–48 h of life	91.8%	51.4%	59.9%	91.2%
Sherwin 2008 [64]	Mixed population: 130 culture-negative vs. 34 culture-positive	EOS and LOS	98 ng/mL	7%	99%	50%	86%
		LOS	1.3 ng/mL	43%	88%	75%	65%
Fendler 2008 [61]	78 preterm newborns	LOS	0.99 ng/mL	97.5%	88.9%	97.5%	88.9%
Ucar 2008 [52,60]	Full term and preterm: 36 cases vs. 36 controls	LOS	0.8 ng/mL	Day 0: 86.1%	97.2%	ND	ND
				Day 4: 83.3%	86.1%		
				Day 8: 69.4%	97.2%		
Boo 2008 [65]	Full term and preterm: 87, 18 with confirmed sepsis	EOS and LOS	2 ng/mL	88.9%	65.2%	ND	ND
Çetinkaya 2009 [67]	Preterm infants: 108 group 1 (high probable sepsis), 5 group 2 (probable sepsis), 10 group 3 (possible sepsis), 40 group 4 (no sepsis, control group).	EOS and LOS	0.5 mg/dL	74.8%	100%	100%	56.3%
Groselj-Grenc 2009 [68]	17 neonates with SIRS vs. 29 controls	LOS	2.28 μg/L	82%	48%	33%	90%
Canpolat 2011 [74]	74 preterm infants with history of pPROM: 32 infected, 42 uninfected	EOS	1.74 ng/mL	76%	85%	ND	ND
Cekmez 2011 [77]	Full term or near term (>34 wks): 62 cases vs. 43 controls	LOS	2.8 ng/mL	86%	81%	ND	ND
Altunhan 2011 [75]	Full term and preterm: Group 1: 171 suspected sepsis Group 2: 89 control group	EOS	At birth 0.59 ng/mL	48.7%	68.6%	48.7%	68.5%
			At 24 h of life 5.38 ng/mL	83.3%	88.6%	83.3%	88.5%
Naher 2011 [76]	Full term and preterm: 10 highly probable sepsis, 11 probable sepsis, 19 possible sepsis, 10 no sepsis	ND	0.5 ng/mL	65%	90%	96.3%	39.1%
Bohnhorst 2012 [78]	Full term and preterm: 58 proven infected, 112 unproven	LOS	0.7 ng/mL	98.3%	65.2%	58.8%	98.6%
Abdollahi 2012 [81]	Full term and preterm: 30- proven EOS -19 clinical EOS -16 negative infectious status -30 uncertain infectious status	EOS	At 12–24 h ≥1.7 ng/mL	76.6%	78.2%	93%	72%
			At 36–48 h ≥4.7 ng/mL	72%	80.4%	76%	70%
Adib 2012 [79]	Full term and preterm: 20 confirmed sepsis vs. 49 clinical sepsis vs. 18 controls	EOS and LOS	1.1 ng/ml	70%	80%	80%	75%
Auriti 2012 [130]	Preterm: 697 controls vs. 65 infected	LOS	0.5 ng/mL	88%	54%	ND	ND
			1 ng/mL	77%	69%		
			2.4 ng/mL	60%	80%		
Ertuğrul 2013 [82]	Premature infants	LOS	ND	91.7%	75%	81.5%	88.2%
Steinberger 2014 [16]	Preterm infants with risk factors for EOS: 30 infected, 188 uninfected	EOS	0.235 mcg/L	78.6%	86.3%	46.8%	96.3%
Park 2014 [83]	Full term and preterm: 18 confirmed sepsis, 56 suspected sepsis, 81 mild infection, 114 controls	ND	0.5 mg/L	88.9%	58.2%	13.2%	98.6%
			1 mg/L	72.2%	69.3%	14.4%	97.2%
Al-Zaharani 2015 [88]	Full-term and preterm: 34 proven EOS, 37 suspected EOS, 29 no EOS	EOS	1.7 ng/mL	72.5%	90%	93.5%	71%
Çelik 2015 [89]	Full-term and preterm: 40 proven sepsis, 76 clinical sepsis, 111 control	EOS and LOS	0.44 ng/dL	75%	86%	60.4%	89.3%
AbdelMohsen 2015 [90]	Full-term and preterm: 35 cases vs. 35 controls	EOS	1.1 pg/mL	80%	85.7%	84.8%	81.1%
Yang 2016 [94]	Full term and preterm: 60 cases and 60 controls	LOS	0.156 μg/L	61.4%	95.1%	93.1%	69.6%
Ozdemir 2016 [95]	Full-term: 29 EOS vs. 40 controls	EOS	2.25 ng/mL	67%	67%	84%	59%
Abd Elmouttaleb 2016 [96]	Gestational age 36–40 wks: 50 cases vs. 30 controls	EOS	2 ng/mL	76.3%	78.2%	65.9%	89.3%
He 2017 [98]	Preterm (>34 wks) and term infants with suspected EOS: 68 infected, 83 uninfected	EOS	differ between different intervals during the first 72 h	86.8%	57.8%	62.8%	84.2%
Montaldo 2017 [100]	Preterm (<34 wks gestational age): 32 cases vs. 38 controls	EOS	0.9 ng/mL	50%	65%	47%	53%
Chen 2017 [99]	Mixed, term and preterm: 96 EOS vs. 44 Non-infective SIRS vs. 53 healthy controls	EOS	3.35 ng/mL	85.4%	86.4%	ND	ND
Kumar 2019 [104]	Mixed, term and preterm: 41 cases vs. 41 controls	EOS and LOS	0.2 ng/mL	97.6%	95.1%	90.2%	97.4%
Rashwan 2019 [103]	Full-term: 66 probable sepsis vs. 102 proven sepsis (47 EOS, 55 LOS)	EOS and LOS	389 pg/mL	97%	100%	100%	93.7%
Ahmed 2019 [107]	Mixed, term and preterm (birth weight more than 1500 gr): 30 cases vs. 30 controls	EOS	2.3 ng/mL	72.2%	80.9%	61.9%	87.2%
Frerot 2019 [131]	Preterm: 45 cases vs. 131 controls	EOS	0.7 μg/L	69%	70%	ND	ND
Stoicescu 2019 [108]	Mixed, term and preterm: 37 cases vs. 49 controls	EOS and LOS	All patients: 0.51 ng/mL	56.4%	42.6%	88%	83.3%
			EOS: 0.51 ng/mL	54.8%	40.9%	85%	90%
			LOS: 0.76 ng/mL	71.5%	95.7%	85.7%	91.7%
Wu 2019 [106]	Full-term and preterm 195 cases vs. 100 controls	EOS and LOS	20.14 μg/L	71%	75.38%	ND	ND
Iskandar 2019 [132]	Mixed, term and preterm: 35 cases vs. 16 controls	EOS and LOS	ND	68.9%	62.5%	80%	47.6%
Morad 2020 [113]	Full term and preterm: 50 neonates with clinically suspected sepsis (31 positive cultures)	EOS and LOS	0.5 ng/mL	97.6%	89%	97.6%	88.9%
Khater 2020 [111]	Mixed, term and preterm: 40 proved sepsis vs. 50 suspected sepsis vs. 30 controls	EOS and LOS	5.6 ng/mL	90%	69%	55%	95%
Yang 2020 [114]	152 preterm (>34 wks) and term infants at risk for EOS: 76 infected, 76 uninfected	EOS	based on concentrations detected at up to 72 h after birth	72.4%	71.1%	56.9%	72.2%
Stocker 2021 [133]	Neonates born after 34 wks: 1678 (553 no sepsis, 952 uncertain, 147 probable, 26 proven)	EOS	2.8 ng/L	100%	ND	ND	ND
Habib 2021 [134]	Full term and preterm: 171 suspected sepsis (86 confirmed by positive cultures)	EOS and LOS	0.5 ng/mL	97.7%	70.6%	77.1%	96.8%
Tang 2022 [115]	Full term and preterm 169	EOS and LOS	27 μg/L	75%	95%	33%	99%

Table Legend: EOS (early onset sepsis); LOS (late onset sepsis); PPV (positive predictive value); NPV (negative predictive value); wks (weeks); GA (gestational age); ND (not declared); pPROM (Preterm Premature Rupture of Membranes).

**Table 3:** Main studies on interleukin-6 (IL-6) accuracy for the diagnosis of neonatal sepsis.

Reference	Population	EOS or LOS	Cut-off Value	Sensitivity	Specificity	PPV	NPV
Messer 1996 [157]	Mixed population, preterm and full-term: 71 infected, 217 uninfected	EOS	100 pg/mL	83.3%	90.3%	ND	ND
Lehrnbecher 1996 [158]	Mixed population, preterm and full-term: 13 infected, 33 uninfected	EOS	150 pg/mL	69%	91%	ND	ND
Smulian 1997 [159]	23 preterm and term infants with suspected EOS: 8 infected, 15 uninfected	EOS	7 pg/mL	88.5%	66.6%	58.8%	91.0%
Panero 1997 [160]	60 NICU preterm and term infants: 13 infected, 47 uninfected	EOS	200 pg/mL	38%	70%	26%	80%
Berner 1998 [161]	Preterm and term infants: 16 infected, 43 uninfected, 35 healthy controls	EOS and LOS	100 pg/mL	87%	93%	76%	97%
Smulian 1999 [162]	Preterm infants: 14 infected, 14 uninfected	EOS	25 pg/mL	92.9%	92.9%	92.9%	92.9%
Silveira and Procianoy 1999 [163]	Newborns with suspected sepsis: 66 infected, 51 uninfected	EOS and LOS	32 pg/mL	90%	43%	67.4%	78.6%
Kashlan 2000 [164]	Very preterm infants (<32 wks GA): 21 infected, 22 uninfected	EOS	100 pg/mL	80%	90%	89%	83
Døllner 2001 [165]	Mixed population, preterm and full-term: 52 infected vs. 33 uninfected	EOS	33.0 pg/mL	84%	70%	ND	ND
Krueger 2001 [166]	Mixed population, preterm and full-term: 40 infected vs. 37 uninfected	EOS	80 pg/mL	96%	94%	ND	ND
Santana 2001 [167]	Mixed population, preterm and full-term: 10 infected, 11 uninfected, 10 healthy controls	EOS	100.8 pg/mL	50%	87%	31%	66%
Martin 2001 [168]	Preterm and term infants with suspected sepsis: 20 infected, 12 uninfected	EOS	30 pg/mL	63%	71%	ND	ND
Hatzidaki 2005 [169]	58 preterm neonates Born to mothers with pPROM: 20 infected, 38 uninfected	EOS	108.5 pg/mL	95%	100%	100	97.4%
			55 pg/mL	90%	97.4%	94.7	94.9%
Gharehbaghi 2008 [170]	Preterm infants born to mothers with PROM: 17 infected, 18 uninfected	EOS	20 pg/mL	46%	85%	88%	39%
Bender 2008 [171]	Preterm and term infants: 29 infected, 94 uninfected	EOS	250 pg/mL	59%	94%	76%	88%
Labenne 2011 [172]	Preterm infants with a suspected diagnosis of EOS: 31 infected, 182 uninfected	EOS	300 pg/mL	87.1%	82%	ND	97.3%
Cernada 2011 [173]	Preterm and term infants with risk factors for EOS: 10 infected, 118 uninfected	EOS	255.87 pg/mL	90%	87.4%	37.5%	99%
Cobo 2013 [174]	Preterm infants born to mothers with pPROM: 12 infected, 164 uninfected	EOS	38 pg/mL	83%	82%	30%	98.1%
Hofer 2013 [175]	Preterm infants at risk of bacterial infection: 32 cases vs. 144 controls	EOS	11.1 pg/mL	815	75%	ND	ND
Cetin 2014 [176]	Preterm infants born to mothers with pPROM: 10 cases vs. 30 controls	ND	11 pg/mL	90%	63.3%	45%	95%
Ebenebe 2019 [177]	Preterm infants (birth weight < 2000 g): 67 cases vs. 115 controls	EOS	40 pg/mL	75%	72.8%	14%	98%

Table Legend: EOS (early onset sepsis); LOS (late onset sepsis); PPV (positive predictive value); NPV (negative predictive value); wks (weeks); GA (gestational age); ND (not declared); pPROM (Preterm Premature Rupture of Membranes).

**Table 4:** Main studies on presepsin accuracy for diagnosis of neonatal sepsis.

Reference	Population	EOS or LOS	Cut-off Value	Sensitivity	Specificity	PPV	NPV
Poggi 2015 [192]	Preterm (≤32 wks of GA): 19 LOS vs. 21 controls	LOS	885 ng/L	94%	100%	100%	95%
Mussap 2015 [193]	Mixed, term and preterm: group A (25 bacterial sepsis), group B (15 SIRS, with no localizing source of bacterial infection), group C (25 non-infected)	EOS and LOS	548 ng/L	100%	81.2%	ND	ND
			600 ng/L	97.5%	100%		
Stojewska 2015 [194]	Mixed, term and preterm: 41 septics, 37 with severe local infections without bacteremia, 16 without infections, but with clinical symptoms suggesting infection and perinatal risk factors and 30 control	EOS and LOS	1066 pg/mL	63.4%	89.2%	ND	ND
Topcuoglu 2015 [195]	Preterm (≤32 wks of GA): 42 LOS vs. 40 controls	LOS	800.5 pg/mL	67%	100%	100%	74%
Abdel Motalib 2015 [196]	Mixed, term and preterm: 28 cases vs. 34 controls	EOS	672 pg/mL	97%	98%	96%	92%
Osman 2015 [197]	Full term neonate: 40 cases vs. 15 controls	EOS and LOS	875 pg/mL	95.7%	87.5%	ND	ND
Xiao 2017 [198]	Mixed, term and preterm: 42 hematosepsis vs. 54 nonhematosepsis vs. 44 non-infectious vs. 53 healthy controls	EOS and LOS	304.5 ng/mL	95.2%	84.9%	ND	ND
Miyosawa 2018 [199]	Preterm: 13 cases vs. 18 preterm controls vs. 35 term controls	EOS	795 pg/mL	85%	89%	85%	89%
Gad 2020 [200]	Full-term: 31 cases vs. 20 controls	EOS	480 ng/L	96.8%	95%	96.8%	95%
			1400 ng/L	100%	88.5%	55.6%	94.7%
Pietrasanta 2021 [201]	Mixed, term and preterm: 58 “infection” vs. 77 septic vs. 24 septic shock	EOS	Overall: 987.5 pg/mL	72%	87%	57%	93%
			Infection: 687.5 pg/mL	81%	62%	15%	98%
			Sepsis: 1013 pg/mL	84%	92%	45%	98%
			Septic shock: 971.5 pg/mL	92%	86%	18%	100%

Table Legend: EOS (early onset sepsis); LOS (late onset sepsis); PPV (positive predictive value); NPV (negative predictive value); wks (weeks); GA (gestational age); SIRS (systemic inflammatory response syndrome); ND (not declared).

## Data Availability

Not applicable.
